# Rare TREM2 variants associated with Alzheimer’s disease display reduced cell surface expression

**DOI:** 10.1186/s40478-016-0367-7

**Published:** 2016-09-02

**Authors:** Daniel W. Sirkis, Luke W. Bonham, Renan E. Aparicio, Ethan G. Geier, Eliana Marisa Ramos, Qing Wang, Anna Karydas, Zachary A. Miller, Bruce L. Miller, Giovanni Coppola, Jennifer S. Yokoyama

**Affiliations:** 1 Department of Molecular and Cell Biology, Howard Hughes Medical Institute, University of California, Berkeley, Berkeley, CA 94720 USA; 2 Memory and Aging Center, UCSF, Department of Neurology, University of California, San Francisco, 675 Nelson Rising Lane, Suite 190, San Francisco, CA 94158 USA; 3 Department of Psychiatry and Semel Institute for Neuroscience and Human Behavior, The David Geffen School of Medicine at University of California Los Angeles, Los Angeles, CA 90095 USA

**Keywords:** TREM2, Genetics, Alzheimer’s disease, Nasu-Hakola disease

## Abstract

Rare variation in *TREM2* has been associated with greater risk for Alzheimer’s disease (AD). *TREM2* encodes a cell surface receptor expressed on microglia and related cells, and the R47H variant associated with AD appears to affect the ability of TREM2 to bind extracellular ligands. In addition, other rare *TREM2* mutations causing early-onset neurodegeneration are thought to impair cell surface expression. Using a sequence kernel association (SKAT) analysis in two independent AD cohorts, we found significant enrichment of rare *TREM2* variants not previously characterized at the protein level. Heterologous expression of the identified variants showed that novel variants S31F and R47C displayed significantly reduced cell surface expression. In addition, we identified rare variant R136Q in a patient with language-predominant AD that also showed impaired surface expression. The results suggest rare *TREM2* variants enriched in AD may be associated with altered TREM2 function and that AD risk may be conferred, in part, from altered TREM2 surface expression.

**Electronic supplementary material:**

The online version of this article (doi:10.1186/s40478-016-0367-7) contains supplementary material, which is available to authorized users.

## Introduction

Alzheimer’s disease (AD) is a common neurodegenerative disorder that occurs in older adults. Clinically, AD is characterized by a decline in cognitive function including memory, language, and/or visuospatial abilities. Aggregation of amyloid-β and hyperphosphorylated tau, which result in the formation of plaques and neurofibrillary tangles, respectively, represent the pathological hallmarks of AD. In addition to factors contributing to accumulation of amyloid and tau, changes in immune function resulting in increased inflammation are thought to contribute to disease pathogenesis and progression.

Common variants like *APOE* ε4 are the best characterized genetic risk factors associated with AD. However, rare genetic variation, which occurs at <1 % minor allele frequency (MAF) in a given population, is becoming increasingly appreciated for its contribution to neurodegenerative disease. These infrequent variants often have more potent biological effects and can occur in genes encoding proteins intimately linked to underlying protein pathology. Rare variants that confer both risk for [1–5] and protection from [6, 7] different forms of neurodegeneration have been identified, but, due to their low MAF, most of these studies required very large cohorts to confirm the effect of these single variants on disease.

*TREM2* is a widely studied gene known to harbor rare variation that can either cause or contribute to risk for distinct neurodegenerative diseases. Homozygous or compound heterozygous mutations in *TREM2* are known to cause Nasu-Hakola disease (NHD) or an early-onset frontotemporal dementia (FTD)-like syndrome, while rare variation in *TREM2* increases risk for AD, and may also increase risk for FTD, Parkinson’s disease, and amyotrophic lateral sclerosis [8–10]. In the brain, TREM2 is an innate immune system receptor expressed primarily on microglia [11]. It has been implicated in sensing damage signals, promoting microglial survival, and regulating central nervous system inflammation [12–14]. In particular, the R47H variant in *TREM2* has been associated with AD risk in populations of European descent [4, 5], and is thought to alter microglial function [13, 15]. Recent evidence suggests that the R47H variant acts by altering TREM2’s ability to bind lipoproteins and apolipoproteins, which may ultimately prevent microglia from efficiently absorbing amyloid-β-lipoprotein complexes [16].

Assessment of mutation burden can alleviate requirements for large cohorts by accounting for the overall risk contribution of rare and even unique variation observed in the same gene but in different individuals. Gene-based analysis offers the unique advantage of weighing the combined effects of multiple variants (common and/or rare) into a single statistical measure of disease risk [17, 18]. Combining rare variants into a single analysis increases power to detect disease-associated risk in a gene using a relatively small cohort [17]. Furthermore, characterizing distinct rare variants occurring within the same functional domain of a particular protein may offer additional insight into shared pathogenic mechanisms. Of the available gene-based tests, the sequence kernel association test (SKAT) and its variants have proven reliable under multiple cohort sizes and have high mean power when compared to other tests [18–20].

In this study, we assessed deep sequencing data from over 150 genes previously linked to neurodegenerative, neuropsychiatric, and neurodevelopmental phenotypes for rare variant burden contributing to AD. We confirmed that mutation burden in *TREM2* is robustly associated with AD risk in two independent cohorts. We then characterized biochemically a subset of rare TREM2 variants to test whether they alter cell surface expression as a means of assessing their functional significance. Our analysis showed that several of the rare variants identified in AD indeed significantly reduced overall expression as well as cell surface expression of TREM2, suggesting that these variants may reduce protein function and contribute to disease risk.

## Materials and methods

### Participants and clinical assessment

For the discovery genetic analysis, 115 males and 161 females were evaluated at the University of California, San Francisco Memory and Aging Center (UCSF MAC), and had genetic data available for analysis. All participants underwent clinical assessment with an in-person visit at the UCSF MAC that included a neurologic exam, cognitive assessment [21, 22] and medical history. Each participant’s study partner was also interviewed regarding functional abilities. A multidisciplinary team composed of a neurologist, neuropsychologist, and nurse then established clinical diagnoses for cases according to consensus criteria for AD and its subtypes [23, 24]. All healthy controls underwent a similar assessment, including study partner interview, and a consensus team of clinicians then established clinical diagnosis of cognitively normal. Controls in this study had Mini-Mental State Exam (MMSE) [25] scores ≥26 or a Clinical Dementia Rating Scale (CDR) [26] of 0, no participant or informant report of cognitive concerns or decline in the prior year, and no evidence from clinical visit suggesting a neurodegenerative disorder (per team neurologist). Detailed demographic information is included in Table 1. Individuals harboring a known disease mutation or with a family history of neurodegeneration were excluded from the study.

**Table 1:** Study participant characteristics

Cohort	Variable	AD	Control	*P*-value
Discovery (UCSF)	N	31	245	
	Age at Onset / First Visit	77.8 ± 4.5	68.5 ± 8.5	*p* < 0.001
	Sex (M / F)	16 / 15	99 / 146	0.18
	Edu (Years, Mean ± SD)	17.0 ± 3.7	17.3 ± 2.1	0.45
	CDR (Mean ± SD)	0.8 ± 0.3	0.0 ± 0.1	*p* < 0.001
	MMSE (Mean ± SD)	22.2 ± 5.3	29.4 ± 0.8	*p* < 0.001
	*APOE* ε4 dose (0 / 1 / 2)	9 / 17 / 5	190 / 48 / 4	*p* < 0.001
	# Pathological Confirmed AD	12		
Replication (ADSP)	N	2927	2633	
	Age at Onset / First Visit	75.3 ± 8.4	85.5 ± 5.1	*p* < 0.001
	Sex (M / F)	1299 / 1628	1185 / 1448	0.639334
	Edu (Years, Mean ± SD)	NA	NA	NA
	CDR (Mean ± SD)	NA	NA	NA
	MMSE (Mean ± SD)	NA	NA	NA
	*APOE* ε4 dose (0 / 1 / 2)	1660 / 1184 / 83	2239 / 386 / 8	*p* < 0.001
	# Pathological Confirmed AD	1057		

Summary demographic, clinical, and genetic information is shown for the Discovery and Replication Cohorts. Note: three individuals in the UCSF Cohort (all controls) do not currently have *APOE* ε4 genotyped. *M* Male, *F* Female, *Edu* Education, *SD* Standard Deviation, *MMSE* Mini Mental State Examination, *NA* Not Available

Replication analysis was performed on samples from the case–control component of the Alzheimer’s Disease Sequencing Project (ADSP), a Presidential Initiative established to identify new genes and alleles contributing to AD risk, AD protection, and targets for new AD therapies, particularly for late-onset AD. The discovery phase of this project generated whole exome sequencing (WES) data for 10,061 unrelated individuals (*N* = 5,096 cases, *N* = 4,965 controls) from the Alzheimer’s Disease Genetics Consortium and the Cohorts for Heart Aging Research in Genomic Epidemiology consortia, of which 5,560 are included in the replication analysis (see Table 1 for cohort demographics). All cases met criteria for probable or definite AD based on clinical assessment, or had neuropathological features of AD upon brain autopsy. Pathological staging was made according to criteria set forth in Braak and Braak (1995) [27]. Cases received a Braak staging score greater than or equal to 3. All controls were clinically assessed for dementia or had an absence of neuropathological AD features upon autopsy (Braak score of 2 or less). Individuals carrying a known disease mutation were excluded from the analyses. All sample phenotype and demographic data were obtained from dbGAP (study accession phs000572.v6.p4; table accession pht004306.v4.p4.c1).

All participants in both analyses were unrelated white individuals (confirmed by identity-by-descent testing in the replication analysis or self-described for those without GWAS data available). Non-Caucasian individuals were excluded due to the insufficient number of participants and potential for confounding background genetics. All aspects of the study were approved by the UCSF Institutional Review Board and written informed consent was obtained from all participants and surrogates (as per UCSF Institutional Review Board protocol).

### Sequencing

The UCSF cohort was screened using targeted sequencing of more than 150 RefSeq genes previously implicated in neurodegenerative dementia, including the most common causative genes for Mendelian forms of AD and FTD. Exonic regions for these genes were captured using a custom-designed Nimblegen SeqCap EZ Choice (Roche) library and sequenced on an Illumina HiSeq2500 at the UCLA Neuroscience Genomics Core (Los Angeles, CA). Sequence reads were mapped to the GRCh37/hg19 reference genome and variants were interactively joint-called with GATK according to GATK Best Practices recommendations (https://www.broadinstitute.org/gatk/ [28]).

ADSP samples underwent WES at one of three NHGRI funded large-scale sequencing centers at Baylor, the Broad Institute, or Washington University. Whole exome capture was performed using either the Illumina Rapid Capture Exome kit or VCRome v2.1 kit (Nimblegen), and paired-end reads were generated using an Illumina HiSeq 2000. Sequence reads were aligned to the GRCh37 reference genome using the Burrows-Wheeler aligner [29], and variants were jointly called across the entire cohort using Atlas V2 software (Baylor) or GATK (Broad). Variants underwent pipeline-specific quality control prior to merging the variants that were concordant between the two sets of variants. The ADSP also performed initial quality control checks on sample information, phenotypes, and genotype data to ensure that these data were of high quality and suitable for downstream analysis.

### Quality control and post-processing

After joint-calling, variants were filtered according to previously established criteria [30]. Briefly, we kept joint-called variants with genotype quality (GQ) scores greater than 30 and read depth (DP) scores greater than 20. The resulting file was annotated with gene names using the Variant Effect Predictor in Ensembl. The predicted effect of each variant were determined using PolyPhen and SIFT. Prior to analysis, we used PLINK [31] to remove individuals with genotyping rates below 95 %, SNPs (single nucleotide polymorphisms) with genotyping rates below 95, and SNPs with a MAF greater than 1 %. Gene SNP sets were created from exonic SNPs classified as missense and nonsense variants. For our replication analysis, we created SNP sets using the same genes that were available for study in our discovery cohort.

### Genetic analyses

Following previously published criteria [18], we limited our analyses to gene SNP sets with 4 or more SNPs available. For our discovery analysis we conducted a SKAT analysis in the amnestic AD cohort from UCSF. Our replication analysis in the ADSP amnestic AD cohort used the same testing parameters and techniques. We repeated the aforementioned analysis in subset of the ADSP cohort that had pathologically confirmed AD. Finally, to test whether rare variation in *TREM2* is associated with clinically heterogeneous AD, we ran a SKAT analysis in a subset of the cohort which included amnestic AD, early-onset AD, executive (frontal) AD, and the logopenic variant of primary progressive aphasia (lvPPA).

### Antibodies

The HA.11 monoclonal antibody used to detect HA-TREM2 was from Covance, and the clathrin heavy chain monoclonal antibody was from BD Transduction Laboratories.

### Molecular biology

The human TREM2 cDNA was obtained from R&D Systems, amplified by PCR and inserted into the pEGFP-N1 vector after first removing the EGFP coding sequence. To facilitate detection of TREM2, an HA epitope tag and linker sequence identical to that used in Kleinberger et al. [14], were inserted after the TREM2 signal peptide using the Phusion high-fidelity DNA polymerase (NEB) system for site-directed mutagenesis. All TREM2 variants were similarly generated using Phusion, with the HA-TREM2 construct serving as template DNA. All constructs were verified by sequencing at the UC Berkeley DNA Sequencing Facility.

### Cell culture

HEK-293T cells were maintained at the UC Berkeley Cell Culture Facility under standard conditions. Cells were transiently transfected using Lipofectamine 2000 (ThermoFisher) according to the manufacturer’s instructions. Culture medium was typically changed 4 h after transfection, and experiments were carried out the following day.

### Immunoblotting

Cells were harvested on ice by washing with cold PBS followed by lysing in a buffer containing 100 mM NaCl, 10 mM Tris-Cl, pH 7.6, 1 % (v/v) Triton X-100 and *Complete* protease inhibitor cocktail (Roche). Triton-insoluble material was sedimented by centrifugation at 20,000 *g* for 10 min at 4 °C. Supernatants were mixed with 5X SDS-PAGE sample buffer supplemented with DTT, then heated at 55 °C for 10 min prior to running in 4–20 % acrylamide gradient gels (Life Technologies and Bio-Rad). After SDS-PAGE, proteins were transferred onto PVDF membranes (EMD Millipore), blocked in 5 % non-fat milk (dissolved in PBS containing 0.1 % Tween-20), and probed with HA and CHC antibodies at 1:2,500 and 1:10,000, respectively. Blots were developed using enhanced chemiluminescence and imaged on a ChemiDoc digital imager (Bio-Rad). Protein signals were quantified using ImageJ (NIH). For overall TREM2 expression analysis, the TREM2 signals derived from cell lysates were first normalized to the corresponding CHC signal, then calculated as a fraction of the WT signal.

### Cell surface biotinylation

Cell surface biotinylation was carried out in a manner similar to that performed in Kleinberger et al., 2014. Briefly, cells were washed at room temperature (RT) with PBS and labeled with the EZ-Link Sulfo-NHS-SS-Biotin reagent (ThermoFisher) at 1 mg/ml in PBS for 15 min. Cells were then placed on ice, washed with cold Tris-buffered saline to quench the biotin reagent, then washed with cold PBS and finally lysed and clarified as described above. To capture biotinylated proteins, *Strep*-Tactin resin (iba) was added to the clarified lysates and the mixtures rotated at 4 °C for 1 h. The resin was then pelleted and washed multiple times with lysis buffer. Finally, 2X SDS-PAGE sample buffer supplemented with DTT was added to the washed resin, and the samples were vortexed, heated and prepared for immunoblotting as described above. For the analysis of surface-labeled TREM2, we quantified the entire surface-labeled signal (including mature and immature bands) by densitometry and normalized the signal of individual variants to the WT signal.

### Statistical analysis

We used the “SKAT” package [18] in R to conduct all gene-based association tests. The SKAT package allows users to conduct sequence kernel association tests, which are powerful when a portion of the variants in a region are noncausal or variant effects are in different directions. All genetic analyses using in the MAC and ADSP cohorts were completed using R.

Protein expression analyses and plots were completed using Graphpad Prism 6 (La Jolla, CA). Protein expression differences were established with ANOVA tests. We used the Holm-Sidak method for our *post hoc* testing.

## Results

### Cohort descriptions

Two hundred and seventy six individuals (245 healthy controls and 31 individuals with AD) participated in this study’s discovery analysis; 5,560 (2,633 healthy controls and 2,927 individuals with AD) participated in this study’s replication analysis. Detailed cohort characteristics by diagnostic grouping for each cohort are provided in Table 1. In both cohorts, there were significant differences in age by diagnostic grouping and *APOE* ε4 dosage, and no significant differences by sex. As expected, in the discovery cohort, there were significant differences in mean CDR and MMSE score. There was no significant difference in education in the discovery cohort.

#### Rare variation in TREM2 is enriched in amnestic Alzheimer’s disease

### Discovery analysis

After quality control and annotation, 157 gene SNP sets were identified. The identified genes are listed in Additional file 1: List S1. Of these, 43 gene SNP sets had 4 or more eligible SNPs and were included in the aggregate burden analysis. The most significant gene SNP set in our discovery analysis was *TREM2* (*p* = 0.001). After multiple testing correction using the Bonferroni method, the p-value for *TREM2* was 0.04. Summary data for this analysis are shown in Table 2. The variants included in the *TREM2* SNP set and their amino acid coding changes are summarized in Table 3. Full results for all 43 SNP sets are shown in Additional file 1: Table S1.

**Table 2:** Aggregate variant burden analysis in discovery and replication cohorts

Cohort	Gene	Testable SNPs	SNPs tested	MAC	*P*-value	Corrected *P*-value
Discovery (UCSF)	***TREM2***	**19**	**8**	**10**	**1.00**×**10** ^**−3**^	**0.04**
	*SMG6*	38	4	4	3.42×10^−2^	NS
	*ARHGAP27*	17	4	6	0.1	NS
	*HSPA6*	18	6	9	0.15	NS
	*LRRK2*	46	9	10	0.16	NS
	*WDR81*	28	10	12	0.2	NS
	*PLCD3*	16	4	5	0.2	NS
	*MAP1B*	28	7	9	0.22	NS
	*UBAP1*	11	4	4	0.22	NS
	*NPEPPS*	11	4	6	0.23	NS
Replication (ADSP)	***TREM2***	**41**	**24**	**330**	**2.88**×**10** ^**−4**^	**0.02**
	*GYPC*	24	16	31	5.19×10^−3^	NS
	*UBAP2*	123	72	207	0.05	NS
	*PACRG*	20	14	93	0.06	NS
	*ZNF621*	32	17	24	0.06	NS
	*EPHX2*	56	38	284	0.08	NS
	*RPIA*	28	9	11	0.09	NS
	*FYN*	28	13	43	0.1	NS
	*HSPA6*	25	20	89	0.11	NS
	*HSPA4*	59	28	183	0.12	NS
Replication (ADSP - Pathology Confirmed)	***TREM2***	**41**	**16**	**192**	**2.11**×**10** ^**−4**^	**0.01**
	*CLU*	51	19	108	2.78×10^−3^	NS
	*KIF24*	136	54	243	6.00×10^−3^	NS
	*GYPC*	24	8	12	0.02	NS
	*BIN1*	61	16	41	0.03	NS
	*RNF19A*	56	23	48	0.03	NS
	*MR1*	43	18	106	0.04	NS
	*EPHX2*	56	29	181	0.06	NS
	*RPIA*	28	7	9	0.09	NS
	*PACRG*	20	12	58	0.09	NS

Results from discovery and replication burden analyses in SKAT. Genes in bold were significant after multiple testing correction. *SNP* single nucleotide polymorphism, *MAC* minor allele count, *NS* not significant

**Table 3:** *TREM2* SNP-set Characteristics

Cohort	CHR	BP	SNP	Minor Allele (+/− strand)	AD MAF	Control MAF	AA change
Discovery (UCSF)	6	41126395	-	A/T	0	0.002041	E202D
	6	41126619	rs138355759	A/T	0	0.002041	**T223I**
	6	41126801	rs371702633	C/G	0	0.002041	**S162R**
	6	41127605	rs149622783	T/A	0	0.002041	**R136Q**
	6	41127606	-	A/T	0	0.002041	**R136W**
	6	41129004	-	A/T	0	0.002041	**A130S**
	6	41129133	rs142232675	T/A	0	0.002041	**D87N**
	6	41129252	rs75932628	T/A	0.04839	0	**R47H**
Replication (ADSP)	6	41126642	-	C/G	0.000171	0	H215Q
	6	41126701	-	T/A	0.000171	0	A196T
	6	41126801	rs371702633	C/G	0.000342	0	**S162R**
	6	41127543	rs2234255	A/T	0.00103	0	H157Y
	6	41127561	rs79011726	T/A	0.000172	0	**E151K**
	6	41129133	rs142232675	T/A	0.001708	0.0009498	**D87N**
	6	41129195	rs201258663	A/T	0.000171	0	T66M
	6	41129252	rs75932628	T/A	0.008944	0.001716	**R47H**
	6	41129253	rs753325601	A/T	0.000172	0	**R47C**
	6	41129295	rs104894002	A/T	0.000345	0	Q33*
	6	41129300	rs746216516	A/T	0.000173	0	**S31F**
	6	41129309	rs2234252	A/T	0.000173	0	**A28V**
	6	41129313	rs768745050	T/A	0.000173	0	**V27M**
	6	41129345	rs777808487	A/T	0.000177	0	S16F

Detailed results for *TREM2* SNP sets used in discovery and replication analyses. Variants in bold were included in the protein expression experiments. *CHR* Chromosome, *BP* Base Pair, *rsID* Reference SNP Cluster ID, *MAF* Minor Allele Frequency, *AA* Amino Acid

### Replication analysis in clinically diagnosed and pathologically confirmed AD

We started with the same 157 genes available in the discovery analysis. Of these, 65 gene SNP sets had 4 or more eligible SNPs. The most significant gene SNP set in the replication cohort was also *TREM2* (*p* = 2.88x10^−4^). After multiple testing correction using the Bonferroni method, the p-value for *TREM2* was 0.02. The variants included in the *TREM2* SNP set and their amino acid coding changes are summarized in Table 3. There were 1,057 neuropathologically confirmed AD cases in the ADSP cohort and we performed an additional analysis restricted to these cases versus all available ADSP controls. Of the 157 genes from the discovery analysis, 61 gene SNP sets had 4 or more eligible SNPs. *TREM2* was also the most significant gene SNP set in the pathology-confirmed AD replication analysis (*p* = 2.11×10^−4^). After multiple testing correction using the Bonferroni method, the p-value for *TREM2* was 0.01. Summary data for both the clinically diagnosed AD replication cohort and pathologically diagnosed AD cohort are shown in Table 2. Full results for all 65 gene SNP sets in the clinically diagnosed AD cohort are shown in Additional file 1: Table S2. Full results for all 61 gene SNP sets in the pathologically diagnosed AD cohort are shown in Additional file 1: Table S3. Together, these results confirm our initial findings from the discovery analysis that rare variants in the exons of *TREM2* are enriched in amnestic AD.

### Secondary analysis in clinically heterogeneous AD

Following our discovery and replication analyses, we conducted an exploratory analysis to identify rare variants in *TREM2* in four clinical variants of AD. We limited our analysis to UCSF participants. The aggregate burden p-value for *TREM2* across all AD subtypes was 0.044. Of note, in this analysis we identified an additional R136Q mutation in an individual with lvPPA. In total, we thus identified R136Q in one patient with lvPPA and in one control in the initial discovery analysis of amnestic AD. A summary of all variants discovered in our analyses by diagnostic grouping and cohort is presented in Additional file 1: Table S4.

#### Rare TREM2 variants show altered cell surface expression

**Fig. 1:**
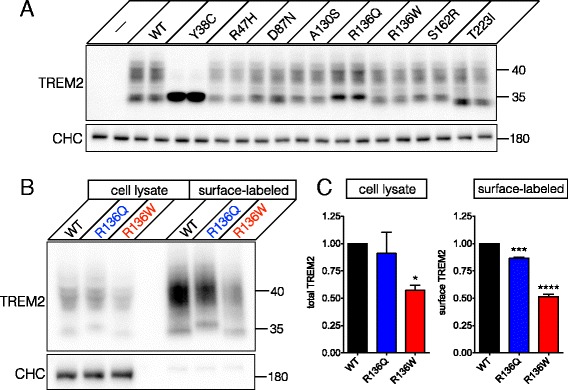
Biochemical characterization of rare TREM2 variants identified at the UCSF Memory and Aging Center. **a** Seven rare TREM2 variants were transiently expressed in HEK-293T cells and compared to cells expressing wild type (WT) TREM2, the Y38C variant or untransfected cells (—). One day after transfection, cells were lysed and the lysates analyzed (in duplicate) by immunoblotting for TREM2 to assess expression, maturation, and electrophoretic mobility. The newly identified variants showed apparently normal maturation, but variant R136Q, identified in a patient with atypical AD, showed slower migration of the immature band. Clathrin heavy chain (CHC) was used as a loading control. **b** and **c** Whole-cell lysate and cell surface biotinylation analysis for variants R136Q and R136W demonstrate significantly reduced overall expression for variant R136W and significantly reduced surface expression for both variants (* *p* < 0.05, *** *p* < 0.001, **** *p* < 0.0001 by ANOVA followed by Holm-Sidak *post hoc* test). CHC was used a loading control for the cell lysates and to confirm the lack of non-specific biotinylation of cytosolic proteins. Results were quantified from three independent experiments

To characterize the rare *TREM2* variants identified in the UCSF cohort at the protein level, we performed site-directed mutagenesis on a human *TREM2* cDNA. We restricted our analysis to coding changes affecting the canonical splice variant of TREM2. For the discovery cohort analyses in amnestic and heterogeneous AD, we generated seven point variants, including those we identified in amnestic AD (R47H), atypical AD / lvPPA and controls (R136Q) as well as those identified specifically in controls (D87N, A130S, R136W, S162R and T223I). As an internal control for our analyses, we also generated the Y38C variant involved in early-onset frontotemporal dementia (FTD) [32], which is known to be defective for protein maturation. These variants were transfected into HEK-293T cells and their expression analyzed by immunoblotting. All variants other than Y38C showed apparently normal protein maturation (Fig. 1a). Interestingly, the immature bands of variants R136Q and T223I showed slightly altered migration by SDS-PAGE, with R136Q migrating slightly slower and T223I slightly faster. Although variant R136Q occasionally showed accumulation of its immature form (Fig. 1a), we did not observe this effect consistently (Fig. 1b). Because we identified R136Q in a patient with atypical AD (as well as in one control), and variants R136Q and R136W have both been observed in AD cases in other studies [2, 4, 5], we further characterized these variants. Of note, R136W has been suggested in unpublished work [33] to show reduced cell surface expression. Thus, we performed cell surface biotinylation on cells expressing these variants. We observed a modest but statistically significant reduction in surface expression for variant R136Q (Fig. 1b, c). In addition, we observed an even larger defect for R136W, highlighting the importance of residue Arg 136 for normal TREM2 surface expression levels. Analysis of the overall expression level of these variants in whole-cell lysates indicated that R136W was significantly reduced (Fig. 1b, c). Mutation of residue Arg 136 thus appears capable of altering both cell surface and overall TREM2 expression.

**Fig. 2:**
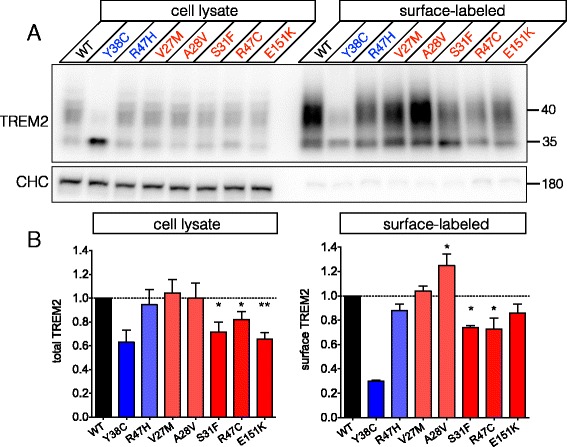
Biochemical analysis of additional rare TREM2 variants identified in the Alzheimer’s Disease Sequencing Project (ADSP) replication cohort. **a** and **b** Five additional rare TREM2 variants identified in patients with AD were analyzed as in Fig. 1. All variants showed normal maturation and mobility by immunoblot analysis. CHC was used as a loading control for the cell lysates and to control for non-specific biotinylation (as above). Whole-cell lysate and cell surface biotinylation analysis indicated that variants S31F, R47C and E151K showed significantly reduced overall expression, while S31F and R47C showed significantly reduced surface expression. Variant A28V specifically showed increased surface expression. Variants Y38C and R47H were used, respectively, as internal controls for severe or modest reductions in cell surface expression (* *p* < 0.05, ** *p* < 0.01 by ANOVA followed by Holm-Sidak *post hoc* test). Results were quantified from 3–4 independent experiments for each variant

We next characterized selected variants identified in the replication cohort, all of which were identified in AD cases. We focused on variants localizing to the extracellular domain, as this is the region affected by the R47H variant as well as many of the NHD-causing mutations in *TREM2*. As with the initial set of variants characterized, we observed apparently normal protein maturation in the five variants studied (V27M, A28V, S31F, R47C and E151K; Fig. 2a, left). However, we observed significant reductions in overall expression as well as cell surface expression for variants S31F and R47C (Fig. 2a, b). Variant E151K showed reduced overall expression, but its trend toward reduced surface expression did not reach significance. To our knowledge, variant S31F has not been previously reported as a risk variant in AD and thus is a novel *TREM2* variant for the field. Variant R47C also appears to be a novel variant and was found in a patient with pathologically confirmed AD (Braak stage 6). Interestingly, R47C affects the same arginine residue altered in the R47H variant. Finally, variant A28V showed a significant increase in cell surface expression.

## Discussion

We confirmed association of aggregate rare variation in *TREM2* with AD in two independent cohorts, including in a subset of individuals with pathologically confirmed AD. Two of the variants identified in AD, S31F and R47C, have not, to our knowledge, been described before. In addition, the R136Q variant identified in an atypical form of AD has not been previously characterized at the protein level. Using heterologous expression, we found that these three variants show a significant reduction in cell surface expression relative to WT TREM2.

Rare homozygous or compound heterozygous mutations in *TREM2* cause NHD or an early-onset FTD syndrome without bone involvement [8, 34]. These include missense mutations such as Y38C, T66M and D86V that occur within the Ig-like domain of TREM2 [32, 35, 36]. Variants Y38C and T66M have been shown to have impaired cell surface expression [14, 37], and we now demonstrate that the novel variants S31F and R47C, which also localize to the Ig-like domain, show reduced surface expression. Thus, it is possible that modestly reduced TREM2 cell surface expression in heterozygotes increases risk for late-onset neurodegeneration, while severely reduced surface expression in homozygotes leads to early-onset FTD or NHD. By extension, we hypothesize that homozygous carriers of S31F, R47C or R136Q, if identified, might be at greater risk for AD neurodegeneration, relative to heterozygotes. In contrast to the above variants, variant A28V, which was also identified in an AD case, showed significantly increased surface expression. It is thus currently unclear if this variant is impaired in another way (e.g., defective ligand binding) or if it contributes risk for disease.

In all of our surface expression analyses, we observed that the immature form of TREM2 was capable of reaching the cell surface. Although this was reported previously for disease-causing variants Y38C and T66M, it was not observed for the WT protein [14]. We speculate that this discrepancy may be due to the different expression systems used (transient expression in this paper vs. stable expression in [14]). Importantly, however, we confirmed the strong reduction in surface expression for variant Y38C that was reported previously in Kleinberger et al. [14] and Park et al. [37], indicating the suitability of our expression method for cell surface labeling.

*TREM2* variant R136Q was identified in one patient with a language-predominant form of AD, the logopenic variant of primary progressive aphasia (lvPPA, [24]). We also identified R136Q and R136W in one control each, underscoring the point that these variants do not appear to be causative for disease. However, others have previously reported both of these variants in amnestic AD [2, 5], and they have MAFs of 0.001278 and 0.0001381, respectively (2 observations in 1,564 alleles and 1 observations in 7,240 alleles, respectively) in the Exome Aggregation Consortium (ExAC) database [38]. The reported MAF of R47H is about 20-fold greater than that reported for R136Q and is consistent with our observation of only one case harboring this variant.

We utilized an aggregate variant burden test implemented in the SKAT program to assess the effects of variation across multiple genes—including *TREM2*—on risk for AD. Advantages of this package include that it makes no assumption about the direction or magnitude of an effect and its ability to account for both a large fraction of noncausal variants and causal variant effects that are in different directions. Some limitations occur when the number of SNPs required in each set results in exclusion of candidate genes. Analysis of larger cohorts with deep resequencing data will be required to expand coverage of rare variation across more genes.

Our finding that some variants do not alter cell-surface expression does not preclude these variants from altering AD risk via other mechanisms. For instance, the V27M and E151K variants did not show significantly reduced surface expression, but may be defective for ligand binding, as has been shown recently for R47H and other variants [16, 39, 40]. Variant A28V, identified in an AD case and showing increased surface expression, may increase risk for disease by adversely affecting ligand binding, or, alternatively, may not affect risk for disease. Future functional studies such as lipoprotein binding and uptake assays will be required to further characterize the effects of the identified variants. We also identified several variants in controls that will require further genetic and functional characterization to determine whether they are likely to alter disease risk. For example, the D87N variant identified in both cases and controls in our cohorts, has recently been shown to display a defect in ligand binding [16] and may thus represent an AD risk variant.

Our study benefits from the analysis of multiple cohorts representing both amnestic and atypical forms of AD, pathological confirmation in a subset of individuals from the replication cohort, and the ability to assess biochemically the effect of select variants on protein expression and cell surface expression. Caveats of the study include a limited number of patients in the discovery cohort—particularly of atypical AD syndromes—and, as mentioned above, the limited scope of genes analyzed.

## Conclusions

In summary, we find that rare variation in *TREM2*, including two variants within the extracellular Ig-like domain, may be associated with risk for AD. Our findings further suggest that impaired overall and cell surface expression of TREM2 may contribute to risk for AD. In addition, since the well-known, AD-associated variant R47H has been proposed to impair TREM2’s ability to bind extracellular ligands [12, 39, 40], it will be interesting to determine in the future whether the variants identified here similarly affect ligand binding. Variants that reduce surface expression without directly impairing ligand binding would be attractive targets for therapeutic intervention that focuses on restoring TREM2 expression at the cell surface.

## Additional file

Additional file 1: Table S1. Full Results for Discovery Analysis. **Table S2.** Full Results for Clinically Diagnosed AD Replication Analysis. **Table S3.** Full Results for Pathologically Diagnosed AD Replication Analysis. **Table S4.** *TREM2* Variants Categorized by Cohort and Phenotype. **List S1.** Genes Available in Discovery Analysis. **Acknowledgment statement for the ADSP**. (DOCX 49 kb)

## Abbreviations

AD: Alzheimer’s disease
ADSP: Alzheimer’s disease sequencing project
CDR: Clinical dementia scale
DP: Read depth
ExAC: Exome Aggregation Consortium
GQ: Genotype quality score
lvPPA: Logopenic variant of primary progressive aphasia
MAF: Minor allele frequency
MMSE: Mini-mental state exam
NHD: Nasu-Hakola disease
SKAT: Sequence kernel association test
SNP: Single nucleotide polymorphism
UCSF MAC: University of California, San Francisco Memory and Aging Center
WES: Whole exome sequencing
WT: Wild type
