# Supplementary material for: Rare TREM2 variants associated with Alzheimer’s disease display reduced cell surface expression
Source: Acta Neuropathol Commun. 2016 Sep 2;4(1):98. doi: 10.1186/s40478-016-0367-7 (PMC5010724; doi:10.1186/s40478-016-0367-7)
Supplement: Additional file 1: Table S1. — Full Results for Discovery Analysis. Table S2. Full Results for Clinically Diagnosed AD Replication Analysis. Table S3. Full Results for Pathologically Diagnosed AD Replication Analysis. Table S4. TREM2 Variants Categorized by Cohort and Phenotype. List S1. Genes Available in Discovery Analysis. Acknowledgment statement for the ADSP. (DOCX 49 kb) [file 40478_2016_367_MOESM1_ESM.docx]

**Additional File 1: SUPPLEMENTARY MATERIAL**

**Table S1: Full Results for Discovery Analysis**

| Gene | Testable SNPs | SNPs Tested | MAC | P-Value |
| --- | --- | --- | --- | --- |
| *TREM2* | 19 | 8 | 10 | 1.00x10^-3^ |
| *SMG6* | 38 | 4 | 4 | 3.42x10^-2^ |
| *ARHGAP27* | 17 | 4 | 6 | 0.10 |
| *HSPA6* | 18 | 6 | 9 | 0.15 |
| *LRRK2* | 46 | 9 | 10 | 0.16 |
| *WDR81* | 28 | 10 | 12 | 0.20 |
| *PLCD3* | 16 | 4 | 5 | 0.20 |
| *MAP1B* | 28 | 7 | 9 | 0.22 |
| *UBAP1* | 11 | 4 | 4 | 0.22 |
| *NPEPPS* | 11 | 4 | 6 | 0.23 |
| *SORT1* | 15 | 5 | 10 | 0.25 |
| *UBQLN1* | 8 | 4 | 7 | 0.26 |
| *GRN* | 22 | 5 | 5 | 0.27 |
| *CLPTM1* | 12 | 4 | 5 | 0.34 |
| *SORL1* | 50 | 13 | 21 | 0.40 |
| *DPH1* | 21 | 7 | 9 | 0.40 |
| *SPPL2C* | 30 | 10 | 15 | 0.40 |
| *EXT2* | 21 | 7 | 11 | 0.43 |
| *PARK2* | 13 | 5 | 8 | 0.47 |
| *SGSM2* | 39 | 10 | 10 | 0.51 |
| *PICALM* | 9 | 5 | 8 | 0.52 |
| *PPP2R1B* | 18 | 6 | 9 | 0.55 |
| *SNCAIP* | 30 | 10 | 13 | 0.55 |
| *RTN4RL1* | 14 | 6 | 14 | 0.59 |
| *UBAP2* | 37 | 12 | 13 | 0.59 |
| *PSEN2* | 13 | 5 | 10 | 0.66 |
| *FMNL1* | 17 | 4 | 4 | 0.69 |
| *HDAC10* | 6 | 4 | 4 | 0.69 |
| *AXIN1* | 25 | 8 | 12 | 0.72 |
| *PINK1* | 16 | 5 | 5 | 0.73 |
| *PLCG1* | 14 | 5 | 5 | 0.73 |
| *MAPT* | 18 | 4 | 6 | 0.76 |
| *SERPINF1* | 21 | 4 | 6 | 0.76 |
| *APOC1* | 6 | 4 | 7 | 0.78 |
| *RORC* | 8 | 4 | 7 | 0.78 |
| *TTBK1* | 14 | 4 | 7 | 0.78 |
| *LRRC37A2* | 12 | 5 | 8 | 0.81 |
| *GOSR2* | 16 | 5 | 9 | 0.83 |
| *SQSTM1* | 18 | 6 | 10 | 0.83 |
| *MR1* | 17 | 7 | 10 | 0.85 |
| *SMYD4* | 13 | 7 | 11 | 0.87 |
| *PVRL2* | 12 | 5 | 12 | 0.88 |
| *KIAA1614* | 26 | 9 | 15 | 0.92 |

Table S1 Legend: Results from discovery SKAT analyses. SNP – single nucleotide polymorphism; MAC – minor allele count.

**Table S2: Full Results for Clinically Diagnosed AD Replication Analysis**

| Gene | Testable SNPs | SNPs Tested | MAC | P-Value |
| --- | --- | --- | --- | --- |
| *TREM2* | 41 | 24 | 330 | 2.88x10^-4^ |
| *GYPC* | 24 | 16 | 31 | 5.19x10^-3^ |
| *UBAP2* | 123 | 72 | 207 | 0.05 |
| *PACRG* | 20 | 14 | 93 | 0.06 |
| *ZNF621* | 32 | 17 | 24 | 0.06 |
| *EPHX2* | 56 | 38 | 284 | 0.08 |
| *RPIA* | 28 | 9 | 11 | 0.09 |
| *FYN* | 28 | 13 | 43 | 0.10 |
| *HSPA6* | 25 | 20 | 89 | 0.11 |
| *HSPA4* | 59 | 28 | 183 | 0.12 |
| *CAMK2B* | 20 | 11 | 13 | 0.12 |
| *SNCAIP* | 108 | 60 | 203 | 0.13 |
| *GSK3B* | 18 | 6 | 8 | 0.15 |
| *MAP1B* | 180 | 100 | 289 | 0.18 |
| *CTNNB1* | 28 | 9 | 22 | 0.19 |
| *NR4A2* | 32 | 14 | 17 | 0.20 |
| *SET* | 10 | 4 | 5 | 0.21 |
| *RNF19A* | 56 | 27 | 61 | 0.26 |
| *RORC* | 40 | 18 | 94 | 0.29 |
| *XPR1* | 44 | 21 | 34 | 0.34 |
| *SORT1* | 56 | 37 | 176 | 0.34 |
| *NCSTN* | 65 | 37 | 130 | 0.35 |
| *UBE2V2* | 7 | 6 | 8 | 0.35 |
| *CDK5* | 12 | 4 | 5 | 0.36 |
| *EIF2AK3* | 59 | 34 | 93 | 0.37 |
| *MARK1* | 52 | 26 | 56 | 0.38 |
| *CCR2* | 42 | 21 | 41 | 0.38 |
| *UBQLN1* | 33 | 14 | 104 | 0.39 |
| *UBE2D4* | 10 | 5 | 7 | 0.40 |
| *PARK7* | 20 | 13 | 26 | 0.41 |
| *SLC25A38* | 33 | 22 | 78 | 0.42 |
| *PARK2* | 59 | 36 | 177 | 0.43 |
| *CCR8* | 32 | 21 | 48 | 0.43 |
| *MR1* | 43 | 24 | 183 | 0.44 |
| *UCHL1* | 19 | 10 | 31 | 0.44 |
| *MOBP* | 9 | 8 | 8 | 0.44 |
| *TARDBP* | 11 | 4 | 12 | 0.45 |
| *THNSL2* | 60 | 33 | 178 | 0.45 |
| *CASP3* | 21 | 12 | 32 | 0.49 |
| *UBAP1* | 38 | 17 | 32 | 0.49 |
| *PPP3CA* | 19 | 9 | 13 | 0.51 |
| *TEX37* | 22 | 14 | 201 | 0.51 |
| *KIF24* | 136 | 75 | 378 | 0.54 |
| *SQSTM1* | 41 | 26 | 98 | 0.56 |
| *THSD7A* | 177 | 99 | 199 | 0.56 |
| *NUDT6* | 40 | 26 | 74 | 0.56 |
| *C5orf45* | 45 | 22 | 94 | 0.61 |
| *FGF2* | 11 | 5 | 5 | 0.61 |
| *PPP3CC* | 40 | 24 | 123 | 0.62 |
| *BIN1* | 61 | 21 | 68 | 0.64 |
| *CDKL3* | 39 | 23 | 70 | 0.66 |
| *TTBK1* | 96 | 41 | 231 | 0.69 |
| *COPA* | 86 | 44 | 67 | 0.72 |
| *PSEN2* | 54 | 35 | 135 | 0.74 |
| *STX6* | 23 | 12 | 20 | 0.77 |
| *OMA1* | 53 | 34 | 261 | 0.78 |
| *PINK1* | 66 | 38 | 108 | 0.80 |
| *CLU* | 51 | 25 | 190 | 0.82 |
| *PRKACG* | 31 | 20 | 26 | 0.84 |
| *KIAA1614* | 143 | 84 | 425 | 0.85 |
| *HSPA1L* | 64 | 29 | 122 | 0.87 |
| *DAB1* | 52 | 28 | 50 | 0.89 |
| *TMEM106B* | 14 | 9 | 12 | 0.89 |
| *PRKACB* | 28 | 14 | 17 | 0.95 |
| *CHMP2B* | 24 | 15 | 32 | 0.98 |

Table S2 Legend: Results from clinically diagnosed replication SKAT analyses. SNP – single nucleotide polymorphism; MAC – minor allele count.

**Table S3: Full Results for Pathologically Diagnosed AD Replication Analysis**

| Gene | Testable SNPs | SNPs Tested | MAC | P-Value |
| --- | --- | --- | --- | --- |
| *TREM2* | 41 | 16 | 192 | 2.11x10^-4^ |
| *CLU* | 51 | 19 | 108 | 2.78x10^-3^ |
| *KIF24* | 136 | 54 | 243 | 0.01 |
| *GYPC* | 24 | 8 | 12 | 0.02 |
| *BIN1* | 61 | 16 | 41 | 0.03 |
| *RNF19A* | 56 | 23 | 48 | 0.03 |
| *MR1* | 43 | 18 | 106 | 0.04 |
| *EPHX2* | 56 | 29 | 181 | 0.06 |
| *RPIA* | 28 | 7 | 9 | 0.09 |
| *PACRG* | 20 | 12 | 58 | 0.09 |
| *CDKL3* | 39 | 15 | 38 | 0.10 |
| *CCR8* | 32 | 14 | 27 | 0.10 |
| *SNCAIP* | 108 | 43 | 130 | 0.10 |
| *UBAP2* | 123 | 52 | 124 | 0.11 |
| *HSPA4* | 59 | 22 | 116 | 0.12 |
| *MAP1B* | 180 | 75 | 182 | 0.13 |
| *TEX37* | 22 | 12 | 116 | 0.14 |
| *EIF2AK3* | 59 | 24 | 55 | 0.15 |
| *NCSTN* | 65 | 31 | 96 | 0.16 |
| *CASP3* | 21 | 6 | 17 | 0.18 |
| *THNSL2* | 60 | 24 | 101 | 0.19 |
| *CHMP2B* | 24 | 12 | 18 | 0.21 |
| *SQSTM1* | 41 | 18 | 59 | 0.21 |
| *OMA1* | 53 | 28 | 165 | 0.22 |
| *C5orf45* | 45 | 16 | 60 | 0.24 |
| *CTNNB1* | 28 | 8 | 15 | 0.25 |
| *UBQLN1* | 33 | 10 | 66 | 0.26 |
| *THSD7A* | 177 | 70 | 117 | 0.30 |
| *CAMK2B* | 20 | 8 | 8 | 0.31 |
| *SORT1* | 56 | 31 | 114 | 0.32 |
| *HSPA1L* | 64 | 23 | 75 | 0.35 |
| *PARK2* | 59 | 30 | 111 | 0.35 |
| *TTBK1* | 96 | 28 | 141 | 0.35 |
| *HSPA6* | 25 | 17 | 55 | 0.36 |
| *FYN* | 28 | 8 | 32 | 0.37 |
| *NUDT6* | 40 | 19 | 48 | 0.39 |
| *UBAP1* | 38 | 9 | 15 | 0.39 |
| *PARK7* | 20 | 11 | 19 | 0.41 |
| *UBE2D4* | 10 | 4 | 6 | 0.43 |
| *PRKACB* | 28 | 7 | 10 | 0.43 |
| *CCR2* | 42 | 14 | 27 | 0.45 |
| *PPP3CC* | 40 | 17 | 79 | 0.46 |
| *SLC25A38* | 33 | 20 | 49 | 0.47 |
| *PSEN2* | 54 | 25 | 90 | 0.47 |
| *PPP3CA* | 19 | 5 | 6 | 0.50 |
| *TMEM106B* | 14 | 5 | 6 | 0.50 |
| *UCHL1* | 19 | 4 | 17 | 0.52 |
| *UBE2V2* | 7 | 5 | 7 | 0.54 |
| *KIAA1614* | 143 | 65 | 263 | 0.55 |
| *GSK3B* | 18 | 4 | 4 | 0.55 |
| *DAB1* | 52 | 23 | 35 | 0.56 |
| *STX6* | 23 | 7 | 12 | 0.59 |
| *MARK1* | 52 | 20 | 31 | 0.61 |
| *XPR1* | 44 | 17 | 27 | 0.63 |
| *PRKACG* | 31 | 14 | 18 | 0.66 |
| *PINK1* | 66 | 29 | 71 | 0.70 |
| *ZNF621* | 32 | 13 | 14 | 0.71 |
| *RORC* | 40 | 12 | 62 | 0.76 |
| *COPA* | 86 | 33 | 43 | 0.78 |
| *MOBP* | 9 | 4 | 4 | 0.88 |
| *NR4A2* | 32 | 9 | 12 | 0.93 |

Table S3 Legend: Results from pathologically diagnosed replication SKAT analyses. SNP – single nucleotide polymorphism; MAC – minor allele count.

**Table S4: *TREM2* Variants Categorized by Cohort and Phenotype**

| ***TREM2* Variant** | **Cohort** | **Phenotype** |
| --- | --- | --- |
| **A130S** | UCSF | Control |
| **A28V** | ADSP | LOAD |
| **D87N** | UCSF + ADSP | Control in UCSF; LOAD in ADSP |
| **E151K** | ADSP | LOAD |
| **R136Q** | UCSF | Atypical AD and Control |
| **R136W** | UCSF | Control |
| **R47C** | ADSP | LOAD |
| **R47H** | UCSF + ADSP | LOAD |
| **S162R** | UCSF + ADSP | Control in UCSF; LOAD in ADSP |
| **S31F** | ADSP | LOAD |
| **T223I** | UCSF | Control |
| **V27M** | ADSP | LOAD |

Table S4 Legend: *TREM2* variants from all analyses are shown. Each variant is categorized by the cohort in which it was found as well as the phenotype(s) it was associated with.

**List S1: Genes Available in Discovery Analysis**

| Gene Name |
| --- |
| *AC006486.9* |
| *ACBD4* |
| *ACCS* |
| *ANP32A* |
| *ANP32D* |
| *APOC1* |
| *APOC2* |
| *APOC4-APOC2* |
| *APOE* |
| *APP* |
| *ARHGAP27* |
| *AXIN1* |
| *BAG5* |
| *BECN1* |
| *BIN1* |
| *C5orf45* |
| *CAMK2B* |
| *CAMK2G* |
| *CARNS1* |
| *CASP3* |
| *CCL2* |
| *CCR2* |
| *CDK5* |
| *CDK5R1* |
| *CDKL3* |
| *CHMP2B* |
| *CLPTM1* |
| *CLU* |
| *CRHR1* |
| *CSNK1D* |
| *CSNK1E* |
| *CTB-129P6.11* |
| *CTNNB1* |
| *DAB1* |
| *DCAKD* |
| *DPH1* |
| *EIF2AK3* |
| *EWSR1* |
| *EXT2* |
| *FGF2* |
| *FMNL1* |
| *FRAT2* |
| *FUS* |
| *FYN* |
| *GATA3* |
| *GOSR2* |
| *GRN* |
| *GSK3A* |
| *GSK3B* |
| *HDAC10* |
| *HEXIM1* |
| *HEXIM2* |
| *HSPA1L* |
| *HSPA2* |
| *HSPA4* |
| *HSPA6* |
| *HSPA8* |
| *JMJD8* |
| *KANSL1* |
| *KIAA1614* |
| *LCMT1* |
| *LRRC37A* |
| *LRRC37A2* |
| *LRRK2* |
| *MAP1B* |
| *MAPK11* |
| *MAPK12* |
| *MAPK8* |
| *MAPT* |
| *MARK1* |
| *MOBP* |
| *MR1* |
| *NCSTN* |
| *NMT1* |
| *NPEPPS* |
| *NR4A2* |
| *NSF* |
| *NUDT6* |
| *OPTN* |
| *OVCA2* |
| *PARK2* |
| *PARK7* |
| *PFN1* |
| *PICALM* |
| *PIN1* |
| *PINK1* |
| *PLCD3* |
| *PLCG1* |
| *PLEKHM1* |
| *PPME1* |
| *PPP1CA* |
| *PPP1CB* |
| *PPP1CC* |
| *PPP2CB* |
| *PPP2R1A* |
| *PPP2R1B* |
| *PPP3CA* |
| *PPP3CB* |
| *PPP3CC* |
| *PRKACA* |
| *PRKACB* |
| *PRKACG* |
| *PRNP* |
| *PSEN1* |
| *PSEN2* |
| *PVRL2* |
| *RNF19A* |
| *RORC* |
| *RPA1* |
| *RPIA* |
| *RPRML* |
| *RPSA* |
| *RTN4RL1* |
| *SEPT11* |
| *SERPINF1* |
| *SERPINF2* |
| *SET* |
| *SGSM2* |
| *SLC25A38* |
| *SMG6* |
| *SMYD4* |
| *SNCAIP* |
| *SOD1* |
| *SORL1* |
| *SORT1* |
| *SPATA32* |
| *SPPL2C* |
| *SQSTM1* |
| *SRR* |
| *STH* |
| *STUB1* |
| *STX6* |
| *TAF12* |
| *TAF15* |
| *TARDBP* |
| *TBX21* |
| *TEX37* |
| *TMEM106B* |
| *TOMM40* |
| *TREM2* |
| *TSR1* |
| *TTBK1* |
| *UBAP1* |
| *UBAP2* |
| *UBE2D4* |
| *UBE2N* |
| *UBE2V1* |
| *UBE2V2* |
| *UBQLN1* |
| *UCHL1* |
| *USP14* |
| *VAPB* |
| *VCP* |
| *WDR81* |
| *WNT3* |
| *WNT9B* |
| *XPR1* |

List S1: Genes with exonic data available are listed alphabetically.

**Acknowledgment statement for the ADSP**

The Alzheimer’s Disease Sequencing Project (ADSP) is comprised of two Alzheimer’s Disease (AD) genetics consortia and three National Human Genome Research Institute (NHGRI) funded Large Scale Sequencing Centers (LSSC). The two AD genetics consortia are the Alzheimer’s Disease Genetics Consortium (ADGC) funded by NIA (U01 AG032984), and the Cohorts for Heart and Aging Research in Genomic Epidemiology (CHARGE) funded by NIA (R01 AG033193), the National Heart, Lung, and Blood Institute (NHLBI), other National Institute of Health (NIH) institutes and other foreign governmental and non-governmental organizations. The Discovery Phase analysis of sequence data is supported through UF1AG047133 (to Drs. Schellenberg, Farrer, Pericak-Vance, Mayeux, and Haines; U01AG049505 to Dr. Seshadri; U01AG049506 to Dr. Boerwinkle; U01AG049507 to Dr. Wijsman; and U01AG049508 to Dr. Goate.

The ADGC cohorts include: Adult Changes in Thought (ACT), the Alzheimer’s Disease Centers (ADC), the Chicago Health and Aging Project (CHAP), the Memory and Aging Project (MAP), Mayo Clinic (MAYO), Mayo Parkinson’s Disease controls, University of Miami, the Multi-Institutional Research in Alzheimer’s Genetic Epidemiology Study (MIRAGE), the National Cell Repository for Alzheimer’s Disease (NCRAD), the National Institute on Aging Late Onset Alzheimer's Disease Family Study (NIA-LOAD), the Religious Orders Study (ROS), the Texas Alzheimer’s Research and Care Consortium (TARC), Vanderbilt University/Case Western Reserve University (VAN/CWRU), the Washington Heights-Inwood Columbia Aging Project (WHICAP) and the Washington University Sequencing Project (WUSP), the Columbia University Hispanic- Estudio Familiar de Influencia Genetica de Alzheimer (EFIGA), the University of Toronto (UT), and Genetic Differences (GD**)**.

The CHARGE cohorts, with funding provided by 5RC2HL102419 and HL105756, include the following: Atherosclerosis Risk in Communities (ARIC) Study which is carried out as a collaborative study supported by NHLBI contracts (HHSN268201100005C, HHSN268201100006C, HHSN268201100007C, HHSN268201100008C, HHSN268201100009C, HHSN268201100010C, HHSN268201100011C, and HHSN268201100012C), Austrian Stroke Prevention Study (ASPS), Cardiovascular Health Study (CHS), Erasmus Rucphen Family Study (ERF), Framingham Heart Study (FHS), and Rotterdam Study (RS).

The three LSSC are: the Human Genome Sequencing Center at the Baylor College of Medicine (U54 HG003273), the Broad Institute Genome Center (U54HG003067), and the Washington University Genome Institute (U54HG003079).

Biological samples and associated phenotypic data used in primary data analyses were stored at Study Investigators institutions, and at the National Cell Repository for Alzheimer’s Disease (NCRAD, U24AG021886) at Indiana University funded by NIA. Associated Phenotypic Data used in primary and secondary data analyses were provided by Study Investigators, the NIA funded Alzheimer’s Disease Centers (ADCs), and the National Alzheimer’s Coordinating Center (NACC, U01AG016976) and the National Institute on Aging Alzheimer’s Disease Data Storage Site (NIAGADS, U24AG041689) at the University of Pennsylvania, funded by NIA, and at the Database for Genotypes and Phenotypes (dbGaP) funded by NIH. Contributors to the Genetic Analysis Data included Study Investigators on projects that were individually funded by NIA, and other NIH institutes, and by private U.S. organizations, or foreign governmental or nongovernmental organizations.
